# Discrepancy between patient and physician in assessment of global severity in early rheumatoid arthritis

**DOI:** 10.1186/ar3631

**Published:** 2012-02-09

**Authors:** Yuko Kaneko, Masataka Kuwana, Tsutomu Takeuchi

**Affiliations:** 1 Division of Rheumatology, Department of Internal Medicine, Keio University School of Medicine, Japan

## Objective

To evaluate the discrepancy between patient and physician in assessment of global severity in early rheumatoid arthritis (RA) and to explore factors affecting the discrepancy at 1-year since the diagnosis of RA.

## Methods

One hundred nine patients with RA with median disease duration of 4 months were enrolled in this study. The global assessment was performed using 100 mm-visual analog scale (VAS). The difference between patient's and physician's assessment wascalculated by subtracting physician's VAS from patient's VAS, and the difference more than 20 mm was defined as discordant. RA patients were stratified by concordance and discordance of VAS scoring at 1-year after the diagnosis. To clarify the factors affecting the discrepancy, clinical characteristics, disease activity using Disease Activity Score (DAS28) 3-variables, functional status by Health Assessment Questionnaire (HAQ) were compared between patients with concordance and discordance.

## Results

The discordance between patient's and physician's VAS at 1-year was found in 41 patients (37%), consisting of 5 patients whose VAS was better than physicians and 36 patients whose VAS was worse than physicians. Tender joint count, DAS28 3-variables, CRP and HAQ were significantly higher in patients with discordance group where patients rated themselves worse than physicians than in patients with concordance (p < 0.05). HAQ score was correlated with the degree of the difference (R = 0.49, p < 0.05).

## Conclusions

Higher disease activity and higher HAQ score was associated the discordance between patient's and physician's VAS in early RA patients, indicating the possibility of physicians underestimating the patient's global disease severity at 1-year since diagnosis.

